# Environmental health during extreme heat: challenges for the practice of safe physical activity in childhood and adolescence

**DOI:** 10.1590/0102-311XEN131825

**Published:** 2026-02-23

**Authors:** Fábio de Freitas, Caroline Fogagnolo, Raquel Lima Dornfeld, Marcia Regina Vitolo, Maria Ângela Antônio, Maria Ângela Bellomo Brandão

**Affiliations:** 1 Universidade Estadual de Campinas, Campinas, Brasil.; 2 Faculdade de Medicina de Ribeirão Preto, Universidade de São Paulo, Ribeirão Preto, Brasil.; 3 Universidade Federal do Triângulo Mineiro, Uberaba, Brasil.

**Keywords:** Physical Activity, Climate Changes, Children, Adolescents, Obesity, Atividade Física, Mudanças Climáticas, Crianças, Adolescentes, Obesidade, Actividad Física, Cambios Climáticos, Niños, Adolescentes, Obesidad

## Abstract

This essay discusses evidence and evidence-based strategies to ensure the safety, well-being, and continuity of physical activity for children and adolescents during extreme heat. It emphasizes the adaptation of environments, professional training, and equity in public policies. High temperatures and sedentary behaviors are not isolated challenges, and they create a critical scenario for the health of children and adolescents when combined, with impacts ranging from thermal regulation to mental health, motor behavior, and risk of obesity. The increasing frequency of extreme heatwaves imposes additional barriers to the safe practice of physical activity among this population, further exacerbating preexisting inequalities. Intense heat increases the risk of dehydration, heat exhaustion, and cognitive impairments, particularly in children, who show lower heat dissipation efficiency due to specific physiological characteristics. Exposure to high temperatures reduces outdoor physical activity, contributing to the worsening of sedentary behavior and pediatric obesity. In this context, adaptive strategies become urgent. Key measures include rescheduling physical activity to cooler times of the day, promoting and ensuring adequate hydration, creating shaded and green outdoor spaces, and strengthening public policies aimed at mitigating environmental impacts on children’s health. Additionally, training education professionals and adapting school infrastructure are essential to ensure safer and health-promoting environments in the face of climate change.

## Abstract (pt)

Este ensaio discute evidências e estratégias baseadas em evidências para garantir a segurança, o bem-estar e a continuidade da atividade física para crianças e adolescentes em contextos de calor extremo. Enfatiza a adaptação dos ambientes, a formação profissional e a equidade nas políticas públicas. Altas temperaturas e comportamentos sedentários não são desafios isolados. Quando combinados, criam um cenário crítico para a saúde de crianças e adolescentes, com impactos que vão desde a regulação térmica até a saúde mental, o comportamento motor e o risco de obesidade. A frequência crescente de ondas de calor extremo impõe barreiras adicionais à prática de atividade física segura nessa população, exacerbando ainda mais as desigualdades pré-existentes. O calor intenso aumenta o risco de desidratação, exaustão pelo calor e comprometimentos cognitivos, particularmente em crianças, que apresentam menor eficiência de dissipação de calor devido a características fisiológicas específicas. A exposição a altas temperaturas reduz a atividade física ao ar livre, contribuindo para o agravamento do comportamento sedentário e da obesidade pediátrica. Nesse contexto, estratégias adaptativas tornam-se urgentes. Medidas-chave incluem a reprogramação da atividade física para horários mais frescos do dia, a promoção e garantia de hidratação adequada, a criação de espaços ao ar livre com sombra e vegetação, e o fortalecimento de políticas públicas voltadas à mitigação dos impactos ambientais na saúde das crianças. Além disso, a capacitação de profissionais da educação e a adaptação da infraestrutura escolar são essenciais para garantir ambientes mais seguros e saudáveis ​​diante das mudanças climáticas.

## Abstract (es)

Este artículo analiza las pruebas y las estrategias basadas en pruebas para garantizar la seguridad, el bienestar y la continuidad de la actividad física de los niños y adolescentes en contextos de calor extremo. Destaca la importancia de la adaptación de los entornos, la formación profesional y la equidad en las políticas públicas. Las altas temperaturas y los comportamientos sedentarios no son retos aislados. Cuando se combinan, crean un escenario crítico para la salud de los niños y adolescentes, con repercusiones que van desde la regulación térmica hasta la salud mental, el comportamiento motor y el riesgo de obesidad. La creciente frecuencia de las olas de calor extremo impone barreras adicionales a la práctica segura de la actividad física entre esta población, lo que agrava aún más las desigualdades preexistentes. El calor intenso aumenta el riesgo de deshidratación, agotamiento por calor y daños cognitivos, especialmente en los niños, que presentan una menor eficiencia en la disipación del calor debido a sus características fisiológicas específicas. La exposición a altas temperaturas reduce la actividad física al aire libre, lo que contribuye al empeoramiento del sedentarismo y la obesidad infantil. En este contexto, las estrategias de adaptación se vuelven urgentes. Las medidas clave incluyen reprogramar la actividad física para las horas más frescas del día, promover y garantizar una hidratación adecuada, crear espacios al aire libre con sombra y vegetación, y fortalecer las políticas públicas destinadas a mitigar los impactos ambientales en la salud de los niños. Además, la formación de los profesionales de la educación y la adaptación de la infraestructura escolar son esenciales para garantizar entornos más seguros y que promuevan la salud frente al cambio climático.

## Introduction

Extreme weather events, especially heat waves, have become a growing concern on a global scale ^1^ ^,^ ^2^. Previous projections showing that global warming would reach 1.5ºC above pre-industrial levels between 2030 and 2052 have been surpassed by recent weather events ^3^. Data from the World Meteorological Organization (WMO) and the Copernicus Climate Change Service confirm that 2024 marked the first time the global average temperature temporarily exceeded the 1.5ºC threshold ^4^. The persistence of heat waves has direct and cumulative implications on human health, amplifying their adverse effects ^5^.

Extreme heat has been identified as a new type of environmental stress, making it even more challenging to engage in regular physical activity, particularly among vulnerable populations ^6^. The COVID-19 pandemic created a similar situation: the closure of schools and restrictions on the use of outdoor spaces led to a sharp decline in physical activity levels and an increase in sedentary behavior ^7^. However, while the pandemic represented a temporary event, the effects of climate change tend to be progressive and long-lasting.

Climate simulations indicate that the duration of heat waves could quadruple by the 2050s, which reinforces the urgency of multisectoral adaptive strategies in the face of environmental and public health risks ^8^.

An increase in ambient temperature has been associated with a reduction in the practice of outdoor physical activity, regardless of age, gender, race, body mass index (BMI), or length of day. It is estimated that for every increase of 1ºC, there is an average reduction of five minutes a day in moderate to vigorous physical activity ^9^ ^,^ ^10^. Such decline contributes to the strengthening of a cycle of sedentary activities, characterized by increased screen time and less participation in physically active behaviors, a pattern associated with worsening sleep quality, increased chronic stress, and a higher risk of metabolic dysfunctions ^11^. Changes in weather patterns also seem to affect the quality of food, which can further exacerbate metabolic and cardiovascular impacts ^12^.

During heat waves, there is an increase in hospital admissions for causes related to children’s mental health, which suggests that prolonged exposure to high temperatures can compromise emotional regulation and make it challenging to use self-regulation strategies in this age group ^13^.

Heat waves pose an increasing challenge for children and adolescents participating in physical activity, as excessive heat acts as a barrier and requires specific adjustments to mitigate their adverse effects on health and well-being ^14^. This situation illustrates how rising temperatures directly influence the routines and habits of this group, particularly regarding motor behavior and outdoor exposure ^15^.

A concrete example of this change is observed in the measures adopted by schools during heat waves, such as the temporary suspension of physical education classes held outdoors ^16^. These measures are designed to protect students from the risks associated with prolonged exposure to intense heat, including dehydration, thermal exhaustion, and exacerbation of preexisting medical conditions ^17^.

Given the increasing frequency and severity of heat waves, a critical question arises: how to ensure the safe practice of physical activity for children in high-temperature contexts? In this scenario, this study aimed to discuss evidence and evidence-based strategies that ensure the safety, well-being, and continuity of physical activity for children during extreme heat, with an emphasis on adapting environments, professional training, and equity in public policies.

## Heat stress and child health

The increase in global temperature, associated with the intensification of extreme weather events such as heat waves, exposes children and adolescents to an increasing risk of heat stress ^18^. Prolonged exposure to intense heat can compromise physical and mental well-being, negatively affect cognitive performance, and increase the occurrence of heat-related health problems such as dehydration, thermal exhaustion, and heat stroke ^19^ ^,^ ^20^.

In environments with high temperatures, the body’s thermal balance is often compromised during physical activity, as the production of metabolic heat can exceed the thermal dissipation capacity. This imbalance results in elevated core and skin temperatures triggering a series of compensatory physiological responses ^21^ ^,^ ^22^. Among these, peripheral vasodilation, increased heart rate, and stroke volume stand out, culminating in higher cardiac output and the activation of sweat glands as a thermoregulatory mechanism ^23^.

The main mechanisms of thermoregulation involve heat loss via radiation, convection, and evaporation. The progressive increase in body temperature imposes an increasing demand on the cardiovascular system, leading to a reduction in stroke volume and a compensatory increase in heart rate to maintain tissue perfusion and blood homeostasis ^24^.

Children have morphophysiological particularities that make them more vulnerable to heat stress compared to adults ^23^ ^,^ ^25^. Among these characteristics, the lower total muscle mass, which limits the production of efficient energy for thermoregulatory mechanisms, and the higher ratio between body surface area and body mass favor heat gain in environments where air temperature exceeds skin temperature ^25^.

Moreover, children tend to rely more on “dry” thermal dissipation mechanisms, such as conduction and convection, than on evaporation, which is considered the most efficient means of compensating for thermal stress ^26^ ^,^ ^27^. This physiological limitation results in lower sweat rates and lower heat dissipation efficiency, favoring greater thermal retention compared to adults ^28^.

These differences contribute to an increased risk of hyperthermia in children, which are more susceptible to extreme heat ^23^. There is also the reduced awareness of lower body temperature, which makes it difficult to perceive thermal discomfort and leads to the spontaneous adoption of protective behaviors. In this context, caregivers have a crucial role in regulating heat exposure and ensuring safe environments ^27^.

Heat stress also imposes functional consequences, affecting neuromuscular and cognitive performance. The diversion of blood flow from the brain to the skin compromises fine motor function, impairs movements precision, and slows down motor responses, increasing the risk of injuries and accidents during physical activity ^29^ ^,^ ^30^. Notably, prolonged exposure to heat compromises short-term memory, diminishes concentration, and promotes behavioral changes, such as irritability and impulsivity. Such effects have direct implications on school and sports performance of children and adolescents ^31^.

## Challenges of exercising in hot environments

Physical activity is essential in managing obesity, whether in childhood or adolescence, contributing not only to weight reduction but also to the prevention of metabolic, cardiovascular, and psychological comorbidities. Regular physical activity is also associated with enhanced emotional well-being, increased self-esteem, and improved social integration ^32^.

According to the guidelines of the World Health Organization (WHO), children and adolescents should perform at least 60 minutes of moderate to vigorous physical activity daily. However, levels of adherence to this recommendation remain critically low: it is estimated that 85% of girls and 78% of boys do not reach this minimum level of movement ^33^. A study conducted in Sweden found that on warmer days, 5-year-olds had a significant decline in outdoor physical activity levels ^27^. The thermal impact proved to be decisive for sedentary behaviors, with a tendency to seek shade and rest as an adaptive response to the heat-generated discomfort. Under such conditions, the environment, which should foster movement and well-being, functions as a significant barrier to the regular practice of physical activity among children and adolescents. In these contexts, the external space ceases to fulfill its role as a health promoter. It begins to impose restrictions on bodily movement, with greater weight falling on the most vulnerable populations ^34^.

Given this scenario, it is urgent to rethink the design of urban and school spaces. The creation of green and shaded areas encourages active play and provides thermal comfort and can be a viable strategy to mitigate the effects of heat on physical activity. Simple interventions, such as planting trees in schoolyards or installing shading structures, have shown potential to maintain children’s physical activity even during high temperatures ^27^.

In this sense, extreme heat, combined with the scarcity of adequate and accessible spaces for movement, constitutes a significant barrier to promote child health. This scenario is also associated with higher rates of obesity and the expansion of environmental and social inequities ^35^. Ensuring outdoor environments that combine thermal safety and movement opportunities is therefore a strategic and urgent measure to address the contemporary challenges posed by climate change on the health of new generations.

## Impacts of heat and obesity in childhood and adolescence

Childhood obesity is one of the most significant global public health challenges, resulting from a complex interaction between genetic, behavioral, and environmental factors ^36^. Climate change, particularly the rise in global average temperatures and the intensification of heat waves, emerges as an additional environmental factor in this multifaceted scenario that negatively affects physical activity levels among children and adolescents, thereby promoting the progression of obesity ^24^.

The very condition of obesity can amplify the effects of heat stress. Children with overweight have greater heat production and retention due to the physiological properties of adipose tissue, which include lower water content and lower conduction and thermal dissipation capacity compared to muscle tissue, thereby favoring body’s temperature accumulation ^37^ ^,^ ^38^. Such thermal imbalance imposes an additional burden on the cardiovascular system, especially under intense heat, increasing the risk of acute physiological complications ^39^.

Cardiorespiratory fitness can act as a protective factor, attenuating the effects of heat stress. Elevated levels of physical fitness in adults are associated with better core temperature control, greater efficiency in sweating mechanisms, and more effective regulation of cerebral blood flow during heat exposure ^40^ ^,^ ^41^. However, evidence on these effects in children and adolescents is still scarce. Given the trend of increasing global temperatures and reducing levels of physical activity in this population, the ability of young people to adapt physiologically to the new climate scenario remains uncertain.

The development and maintenance of adequate physical activity levels and cardiorespiratory fitness may represent a key strategy for mitigating the impacts of heat stress and increasing children’s resilience in the face of climate change. Understanding this relationship is essential to guide public policies and more effective preventive interventions aimed at promoting healthy habits during childhood ^24^.

## Strategies for safe physical activity in high temperatures

Rethinking the way physical activity is planned and conducted is essential, especially during high temperatures ^23^. Professionals who work directly with this public, such as physical education teachers, must be appropriately trained to identify early signs of thermal exhaustion. Among the most common symptoms are pallor, intense facial flushing, dizziness, headache, nausea, chills, as well as changes in mood and mental status ^18^. The prompt identification of these signs is essential to prevent more severe complications and ensure the safety of children during physical activity in hot environments.

Physical educators and professionals who work with children and adolescents must be able to implement specific safety protocols, ranging from adapting physical effort to broader preventive measures. There are indispensable strategies, such as: modifying practice schedules, prioritizing cooler periods of the day, reducing exercise intensity, and progressively adapting to effort. The use of light clothing, which favors heat dissipation, should also be encouraged ^42^.

Another critical aspect is maintaining proper hydration, before, during, and after activities. Hypohydration, which is characterized by reduced water availability in the body due to excessive fluid and electrolyte loss, is a common condition under heat stress and can compromise both performance and physical safety. To prevent it, regular water intake is recommended throughout the practice: approximately 100 to 250mL every 20 minutes for children between 9 and 12 years old, and between 1 and 1.5 liters per hour for adolescents, provided they are previously hydrated ^30^.

## Public policies and mitigation initiatives

Extreme weather events tend to exacerbate health inequalities, particularly in low-income communities, where existing structural barriers to outdoor physical activity include lack of safety, absence of green spaces, and scarcity of adequate facilities. The prevalence of pediatric obesity is often higher in these areas, and extreme heat shows another obstacle, further discouraging physical activity ^35^.

Mitigating the effects of climate change requires not only actions aimed at reducing greenhouse gas emissions, but also the strengthening of adaptive strategies. The analysis of the 2024 Europe report of the *Lancet* Countdown on health and climate change underscores that epidemiological surveillance and educational strategies are essential to mitigate health risks associated with extreme heat ^43^. Among these, the expansion of green infrastructure, such as urban afforestation and vegetation in public areas, and the formulation of public policies focused on reducing the vulnerability of exposed populations stand out, particularly for children living in areas most affected by heat waves ^44^. Adaptation strategies, such as readjusting physical activity schedules for cooler periods, are essential, however, equity must be considered, since this flexibility of time can be a privilege, not being accessible to all children and adolescents ^43^.

These urban interventions play a dual role: they promote environmental sustainability and improve air quality, and favor the practice of physical activity, contributing to the prevention of childhood obesity ^45^. At the same time, it is crucial to expand access to covered sports courts, particularly in school and community contexts, ensuring environments are minimally protected from direct exposure to intense heat ^46^.

The adaptation of urban and school infrastructure should not be treated as a privilege, but as a commitment to health equity ^47^. Ensuring that all children, have access to safe conditions for movement and play, regardless of their family income or where they live, should be at the heart of policy and urban planning decisions.

Therefore, promoting safe environments for physical activity in adverse weather conditions is a matter of logistical adaptation and of protecting the health and rights of children. This responsibility requires well-prepared professionals, adequate infrastructure, and public policies aligned with the new environmental reality.

## Data Availability

Not applicable.
